# Numerical Investigation of Buckling Behavior of MWCNT-Reinforced Composite Plates

**DOI:** 10.3390/ma18143304

**Published:** 2025-07-14

**Authors:** Jitendra Singh, Ajay Kumar, Barbara Sadowska-Buraczewska, Wojciech Andrzejuk, Danuta Barnat-Hunek

**Affiliations:** 1 Government Engineering College Buxar, Bihar 802103, India; 2 National Institute of Technology Delhi, New Delhi 110036, India; sajaydce@gmail.com; 3 Faculty of Civil Engineering and Environmental Sciences, Bialystok University of Technology, Wiejska St. 45A, 15-351 Bialystok, Poland; barbara.sadowska@pb.edu.pl; 4 Faculty of Technical Sciences, John Paul II University in Biała Podlaska, Sidorska 95/97, 21-500 Biała Podlaska, Poland; w.andrzejuk@dyd.akademiabialska.pl; 5 Faculty of Civil Engineering and Architecture, Lublin University of Technology, Nadbystrzycka St. 40, 20-618 Lublin, Poland

**Keywords:** composite laminates, finite element method, buckling analysis of plate, shear deformation plate theory

## Abstract

The current study demonstrates the buckling properties of composite laminates reinforced with MWCNT fillers using a novel higher-order shear and normal deformation theory (HSNDT), which considers the effect of thickness in its mathematical formulation. The hybrid HSNDT combines polynomial and hyperbolic functions that ensure the parabolic shear stress profile and zero shear stress boundary condition at the upper and lower surface of the plate, hence removing the need for a shear correction factor. The plate is made up of carbon fiber bounded together with polymer resin matrix reinforced with MWCNT fibers. The mechanical properties are homogenized by a Halpin–Tsai scheme. The MATLAB R2019a code was developed in-house for a finite element model using C^0^ continuity nine-node Lagrangian isoparametric shape functions. The geometric nonlinear and linear stiffness matrices are derived using the principle of virtual work. The solution of the eigenvalue problem enables estimation of the critical buckling loads. A convergence study was carried out and model efficiency was corroborated with the existing literature. The model contains only seven degrees of freedom, which significantly reduces computation time, facilitating the comprehensive parametric studies for the buckling stability of the plate.

## 1. Introduction

Composite materials are created by the combination of at least two materials with varying mechanical and physical properties. As compared to their individual components, composite materials exhibit superior material properties, because they tend to minimize the weakness and improvise the strength of its constituent materials. Their advantages include enhanced strength-to-weight ratios and corrosion resistance, making them ideal for engineering applications like automobile, aerospace, biomedical, and marine structures. Since 1960, composites have found extensive application in engineering structures due to their ability to be customized for superior mechanical and chemical properties compared to its constituents. However, delamination is a major challenge in composite material laminates and this can be reduced by the addition of reinforcing MWCNT fibers.

Composite materials are prone to buckling when used as laminates under the action of compressive loading. The equilibrium displacement of these thin and slender laminated structures buckles when the compressive load increases beyond a certain critical load. The anisotropic nature of the composite laminates and additional non-homogeneity due to MWCNT fibers increases the complexity of the microstructure. The Halpin–Tsai (H-T) Model [1] provides a simplified semi-empirical material homogenization scheme to estimate the mechanical properties characterizing the composite material. Nonetheless, there are number of design parameters that present major challenges in conducting the structural analysis of the composite materials. Consequently, many design and analysis techniques for composite structures have been increasingly explored for comprehensive evaluation of their mechanical properties.

Exact three-dimensional (3D) theories [2,3,4,5] directly addressed the full three-dimensional equations of elasticity. And hence, they predicted the displacement and stresses of the plates under external loading with great accuracy. The 3D exact plate theory offers a more accurate representation of plate behavior and the results obtained from these theories can serve as the benchmark for the other research works. The complexity of the solutions and requirement of large computations often limit the application of this theory for simple geometries and loading conditions. The complexity of the three-dimensional equations of elasticity can be reduced either by layerwise partition of the structure or by decreasing the degrees of freedoms of structure into equivalent single layer. In layerwise theories, variables are dependent on the layer and each layer is analyzed separately. Boundary conditions are satisfied at the interface of each layer (Robbins and Reddy [6], Li [7], Rakočević et. al. [8], Goswami and Becker [9]). Computations and accuracy are reduced when compared to 3D exact plate theories. In equivalent single layer (ESL) theories, calculations are limited to mid-plane displacements and all the other displacements are modelled as the function of its thickness coordinates and displacement of the mid-plane. Using ESL theories, computations are reduced drastically.

However, the reliability of the results is reliant on the type of functions employed for modelling.

ESL theories like Kirchhoff’s classical plate [10] theory omits the effect of transverse shear stress completely. This theory produces acceptable outcomes for thin plates while the impact of transverse shear is noticeable for thick and moderately thick plates. As a result, the stresses and displacement calculated using this theory has some errors, like for moderately thick plates, for instance, CLPT (Classical Laminate Plate Theory) overpredicts the buckling. To address this issue, the first-order shear deformation theory (FSDT) is suggested. FSDT, founded on plate theory of Reissner [11] and Mindlin [12], takes an independent field variable for the rotation of the transverse normal vector in order to consider the transverse shear stress effect. However, this theory results in finite transverse shear stress on plate surface. In order to adequately fulfill the traction-free boundary condition at both the upper and lower plate surfaces, a shear correction factor is thus added. However, determining the shear correction factor value for real-world issues is challenging, since it is mostly dependent upon loading circumstances, boundary conditions, stacking scheme, geometry, and complex material characteristics.

Second-order shear deformation theories (Khdeir and Reddy [13], Shahrjerdi et. al. [14]) gave marginally improved results for the stresses and displacement when compared to FSDT. However, the requirement of a shear correction factor still existed, restricting its computational advantages. Third-order shear deformation theories are developed to eliminate the necessity for shear correction factors, accurately account for cross-sectional warping, as well as achieve a realistic representation of the variations in transverse shear strains and stresses. These theories are refined by using cubic polynomial (Shi [15], Aagaah et al. [16], Ferreira et al. [17]) or orthogonal polynomial like Legendre polynomials (Carrera et al. [18], Pagani [19], Verma et al. [20]) and reduce the number of variables to ensure traction free boundary conditions at the upper and lower plate surface and model the flexural behavior of the plate. Some non-polynomial shear deformation functions like trigonometric (Thai and Vo [21], Ferreira et al. [22], Wang and Wu [23]), exponential (Mantari et al. [24], Khorshidi and Fallah [25]), hyperbolic (Meiche et al. [26], Grover et al. [27], Thai et al. [28]), and inverse trigonometric functions (Nguyen et al. [29], Grover et al. [30]) are also utilized for modelling. The infinite differentiability of these functions and peculiar nonlinearity makes them more suitable for capturing the accurate behavior of the transverse shear stresses and strain without increasing the number of additional parameters for calculation. This results in increase in computational efficiency of the model without compromising with the accuracy of the solutions.

The differential equation for the plate under various loading conditions is solved using Fourier series expansions like Navier solutions (Farahmand et. al. [31], Levinson and Cooke [32]) and Levy solutions (Mohammadi et al. [33], Thai et al. [34]), or by using approximate solutions for series of functions like the Galerkin method and Reyleigh–Ritz method (Lam et al. [35], Liew [36]). While analytical methods provide exact solutions, they are often limited to simple geometries and loading conditions. For complex geometries and boundary conditions, various numerical methods are more practical. Additionally, these transverse strains involve normal and transverse shear stresses, which, due to equilibrium considerations, exhibit continuity at every layer interface. These conditions require solutions to satisfy C^1^-continutity conditions. These criteria can be modelled by using layer-independent Murakami’s Zig-Zag Functions (Carrera [37], Brischetto et al. [38]) or by using meshfree methods (Liew et al. [39], Belinha et al. [40]), where a set of scattered nodes is used to approximate the solutions. This makes them particularly useful for problems with complex geometries, large deformations, and moving boundaries. However, the implementation of C^1^-contintuity of displacement and slopes of zig-zag theories or meshless techniques requires use of complex algorithms. Therefore, simple C^0^-continuity finite element solutions were developed and refined for this purpose. Their simplicity and efficacy to model complex geometric, loading and boundary conditions accurately is remarkable.

Lei et al. [41] conducted a buckling analysis on composite plates reinforced with carbon nanotubes (CNT-RC) with functionally graded (FG) properties by employing a set of mesh-free kernel particle functions and FSDT. Shen and Zhu [42] investigate the phenomenon of buckling and post-buckling in nanocomposite plates subjected to uniaxial compression, specifically focusing on the presence of functionally graded (FG) nanotube reinforcements. An investigation by Meng and Gardner [43] explored the stability of circular hollow section (CHS) columns, focusing on both normal and high strength steel through experimental and numerical methods. The deformation behavior of a composite laminated plate reinforced with carbon nanotubes (CNTs) simulations to investigate different types of carbon nanotube (CNT) distributions was examined by Lei et al. [44]. Zhang [45] studied the buckling characteristics of nanocomposite plates featuring polygonal platforms subjected to in-plane loads.

The study conducted by Srivastava and Kumar [46] examined the buckling and post-buckling characteristics of a nanocomposite plate containing randomly oriented carbon nanotubes in magnesium (Mg) under uniaxial compression. Kiani [47] investigated the issue of post-buckling in composite plates that were reinforced with CNTs and exposed to a homogeneous thermal stress. Thai and Kim [48] proposed the utilization of the third-order higher-order shear deformation theory (HSDT) to derive a closed-form solution for the buckling analysis of a thick functionally graded (FG) plate resting on an elastic foundation. Assessments on the free vibration and buckling characteristics of laminated non-rectangular plates reinforced with carbon nanotubes (FG-CNTR) were conducted by Civalek and Avcar [49], who employed a four-noded straight-sided transformation approach. An analytical model involving small-strain, moderate-rotation shell theory in conjunction with a linearly viscoelastic material law was adopted by Liu et al. [50].

Literature reviews showed that numerous shear deformation theories have been developed for modelling the composite laminate. However, for efficient and simplistic modelling of the composite laminates reinforced with MWCNT fibers, there is a need of novel HSDT, which can accurately predict the buckling behavior of MWCNT fiber interaction with composite laminates. The proposed equivalent single layer HSDT is accurate, simple for implementation, and increases the computational speed. The proposed mathematical formulation ensures C^0^-continutity of displacements, which increases its simplicity in formulation and implementation. Inhouse code written in MATLAB (R2019a) is used to write the algorithm of the mathematical formulation for the comprehensive evaluation of the plate buckling behavior of MWCNT-added composite laminate. Excellent convergence has been observed in the critical buckling loads of the model and the obtained results were corroborated with the findings from previous publications and have been found to be accurate. A parametric study has been performed on the model for the comprehensive evaluation of models and to understand the effect of MWCNT fibers on composite laminates.

## 2. Mathematical Formulation

### 2.1. Composite Laminate Geometry

**Figure 1:**
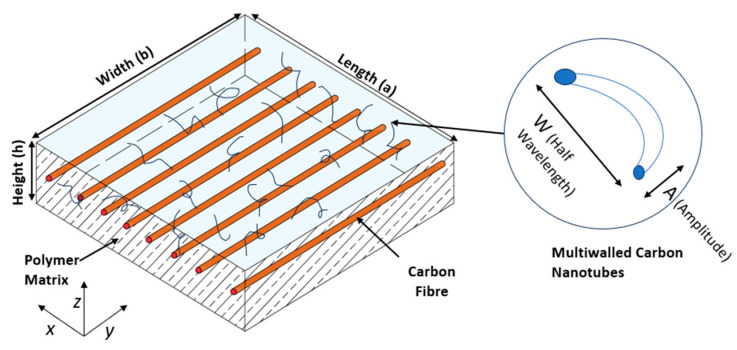
Composite laminated epoxy resin and carbon fiber polymer matrix composite incorporating MWCNT additions within the matrix.

The geometrical parameters of the MWCNT-added laminated composite, including length (a), width (b), and thickness (h), are illustrated in Figure 1. The coordinates *x* and *y* are employed for in-plane placement, whereas *z* is utilized for locating in the thickness direction. The fiber orientation angle is measured from the major axis (*x*-axis) in a clockwise manner. The laminate number and fiber orientation are supplied in order to analyze the composite laminate.

### 2.2. Material Homogenization Scheme

The H-T model governs the mechanical properties characterizing the MWCNT-reinforced polymer matrix, accounting for agglomeration, orientation, and waviness. The mechanical parameters (δ) of the composite lamina are derived from the characteristics of MWCNT reinforcements and the fiber-polymer matrix composite, as outlined in the methodology of Georgantzinos et al. [51]. The elastic modulus characterizing the polymer matrix reinforced with multi-walled carbon nanotubes ($E_{m-cnt}$) is presented below:

(1) $$E_{m-cnt}=E_{m}\times \frac{1+R\delta V_{cnt}}{1-\delta V_{cnt}}$$

In this context, E_m_ represents the elastic modulus of the polymer matrix, V_cnt_ denotes the volume fraction of multi-walled carbon nanotubes (MWCNTs), whereas R corresponds to the ratio of the length to the radius of the MWCNTs. The expression below can be used to determine parameter δ:

(2) $$\delta =\frac{f_{w}f_{a}f_{r}\frac{E_{cnt}}{E_{m}}-1}{f_{w}f_{a}f_{r}\frac{E_{cnt}}{E_{m}}+2R}$$

(3) $$f_{w}=1-\frac{A}{W}$$

Waviness corresponds to parameter f_w_, as delineated in Equation (3), where A denotes the MWCNT bend amplitude, whereas W signifies its half-wavelength. The stochasticity of MWCNT orientation governs the parameter f_r_. If the lamina thickness is greater than MWCNT length, f_r_ = 1/3 indicating slight orderliness; if not, f_r_ = 1/6, which indicates large randomness. MWCNT aggregation is represented in Equation (4) by f_a_, where α and β describe the degree of CNT agglomeration, α is set at 10 and β at 0.9 for this research work.

(4) $$f_{a}=-\alpha {V_{cnt}}^{\beta }$$

The Poisson ratio, υ, corresponding to a polymer matrix reinforced with MWCNT can be considered equivalent to that of the pure polymer matrix, that is, υ_m-cnt_ = υ_m_. Given the uniform distribution of MWCNTs, the polymer matrix added with MWCNT functions is a material with quasi-isotropic properties. The shear modulus characterizing the polymer matrix reinforced with multi-walled carbon nanotubes ($G_{m-cnt}$) is calculated in the following way:

(5) $$G_{m-cnt}=\frac{E_{m-cnt}}{2\left(1+\vartheta_{m-cnt}\right)}$$

(6) $$E_{1}=E_{f}V_{f}+E_{m-cnt}V_{m-cnt}$$

(7) $$\vartheta_{12}=\vartheta_{f}V_{f}+\vartheta_{m-cnt}V_{m-cnt}$$

where the $E_{1}$ and $\vartheta_{12}$ represents the in-plane Young’s modulus and Poisson’s ratio of elasticity. And $E_{f}$, $V_{f}$, and $\vartheta_{f}$ correspond to the Young’s modulus, the volume fraction and Poisson’s ratio of fiber, respectively. Equation (8) can be used to determine additional mechanical properties, where *P* may denote the transverse shear moduli (*G*_13_ and *G_23_*), in-plane shear modulus (*G_12_*), transverse Poisson ratio (*υ_23_*), and transverse modulus of elasticity (*E_2_*) whereas *P_m-cnt_* may refer to material properties of the polymer matrix reinforced with MWCNT. The parameter ξ (reinforcing factor) equals 2 for *E_2_* and ξ equals 1 for υ_23_, *G_12_*, and *G_23_*. Equation (9) is used to ascertain parameter η (experimental factor).

(8) $$\frac{P}{P_{m-cnt}}=\frac{1+\xi \eta V_{f}}{1-\eta V_{f}}$$

(9) $$\eta =\frac{\frac{P_{f}}{P_{m-cnt}}-1}{\frac{P_{f}}{P_{m-cnt}}+\xi }$$

### 2.3. Displacement Field Model

The displacement fields in the current HSDT are delineated as follows:

(10) $$u\left(x,y,z\right)=u_{0}\left(x,y\right)-z\frac{\partial w_{0}}{\partial x}+\psi \left(z\right)\phi_{sx}\left(x,y\right)$$

(11) $$v\left(x,y,z\right)=v_{0}\left(x,y\right)-z\frac{\partial w_{0}}{\partial y}+\psi \left(z\right)\phi_{sx}\left(x,y\right)$$

(12) $$w(x,y,z)=w_{0}\left(x,y\right)$$

Five fundamental nodal unknowns, including u, v, w, ϕ_sx_, and ϕ_sy_, constitute the displacement fields. Additionally, u, v, and w represent the displacements on the mid-plane of the plate along the *x*-, *y*-, and *z*-axes, respectively. In turn, ϕ_sx_ and ϕ_sy_ denote the rotation of the plate’s transverse normal about the *y*-axis and *x*-axis, respectively. The distribution of transverse shear strain over the plate’s thickness is delineated by the form function ψ(z), which combines polynomial and hyperbolic functions.

(13) $$\psi \left(z\right)=\frac{h}{2}tanh\left\{\frac{2z}{h}\right\}-\frac{4z^{3}}{3h^{2}{cosh\left(1\right)}^{2}}$$

The derivatives of out-of-plane first-order provide challenges as the strain expressions will include second-order derivatives, necessitating the enforcement of C^1^ continuity. The C^1^ continuity criteria are intricate and challenging to simulate. Consequently, the out-of-plane derivatives are replaced by supplementary rotating unknowns, θ_bx_ and θ_by_, to guarantee C^0^ continuity in the finite element modeling. The supplementary degrees of freedom are artificially restricted by integrating Constraint Equations (14) and (15) into the computation of the system’s strain energy.

(14) $$\theta_{bx}=\frac{\partial w_{0}}{\partial x}$$

(15) $$\theta_{by}=\frac{\partial w_{0}}{\partial y}$$

The revised displacement fields Equations (14) and (15) can be expressed as follows:

(16) $$u\left(x,y,z\right)=u_{0}\left(x,y\right)-z\theta_{bx}+\psi \left(z\right)\phi_{sx}\left(x,y\right)$$

(17) $$v\left(x,y,z\right)=v_{0}\left(x,y\right)-z\theta_{by}+\psi \left(z\right)\phi_{sx}\left(x,y\right)$$

Consequently, each node at the midplane comprise seven total nodal unknowns, in turn, in the formula below may be used to express the midplane displacement vector:

(18) $$X=\left[u_{0},v_{0},w_{0},\theta_{bx},\theta_{by},\phi_{sx},\phi_{sy}\right]$$

### 2.4. Strain Displacement Relationship

The equations below can be employed to express the strain-displacement relationship, derived from the differentiation of the displacement field equations, is articulated as follows:

(19) $$\in_{xx}=\frac{{\partial u}_{o}}{\partial x}-Z\frac{{\partial \theta }_{bx}}{\partial x}+\psi \left(z\right)\frac{{\partial \phi }_{sx}}{\partial x}$$

(20) $$\in_{xx}=\frac{{\partial u}_{o}}{\partial x}-Z\frac{{\partial \theta }_{bx}}{\partial x}+\psi \left(z\right)\frac{{\partial \phi }_{sx}}{\partial x}$$

(21) $$\gamma_{xy}=\left\{\frac{{\partial u}_{o}}{\partial y}+\frac{{\partial v}_{o}}{\partial x}\right\}-Z\left\{\frac{{\partial \theta }_{bx}}{\partial y}+\frac{{\partial \theta }_{by}}{\partial x}\right\}+\psi \left(z\right)\left\{\frac{{\partial \phi }_{sx}}{\partial y}+\frac{{\partial \phi }_{sy}}{\partial x}\right\}$$

(22) $$\gamma_{xz}=\frac{\partial \psi }{\partial z}\theta_{sx}+\left\{-\theta_{bx}+\frac{\partial w_{0}}{\partial x}\right\}$$

(23) $$\gamma_{xz}=\frac{\partial \psi }{\partial z}\theta_{sy}+\left\{-\theta_{by}+\frac{\partial w_{0}}{\partial y}\right\}$$

These stresses may be expressed as the product of the midplane nodal unknowns and their derivatives, which vary across the plane, ϵ_o_, as follows:

(24) $$\epsilon_{0}=\left\{\begin{array}{c} \frac{{\partial u}_{o}}{\partial x},\frac{{\partial v}_{o}}{\partial y},\frac{{\partial u}_{o}}{\partial y}+\frac{{\partial v}_{o}}{\partial x},\frac{\partial w_{0}}{\partial x},\frac{\partial w_{0}}{\partial y},\frac{{\partial \theta }_{bx}}{\partial x},\frac{{\partial \theta }_{by}}{\partial y},\frac{{\partial \theta }_{bx}}{\partial y}+\frac{{\partial \theta }_{by}}{\partial x},\\ \theta_{bx},\theta_{by},\frac{{\partial \phi }_{sx}}{\partial x},\frac{{\partial \phi }_{sy}}{\partial y},\frac{{\partial \phi }_{sx}}{\partial y}+\frac{{\partial \phi }_{sy}}{\partial x}\;,\theta_{sx},\theta_{sy} \end{array}\right\}$$

### 2.5. Constitutive Equation Ignoring Thickness Stretching (ε_zz_ = 0)

The constitutive equations employed in the analysis of MWCNT-reinforced composite laminates and nonlinear buckling analysis disregard the influence of thickness stretching, with the stress-strain relationship defined by the constitutive relationship presented in Equation (25).

(25) $$\left[\begin{array}{c} \sigma_{xx}\\ \sigma_{yy}\\ \tau_{xy}\\ \tau_{xz}\\ \tau_{yz} \end{array}\right]=\left[\begin{array}{ccccc} Q_{11} & Q_{12} & 0 & 0 & 0\\ Q_{12} & Q_{22} & 0 & 0 & 0\\ 0 & 0 & Q_{33} & 0 & 0\\ 0 & 0 & 0 & Q_{44} & 0\\ 0 & 0 & 0 & 0 & Q_{55} \end{array}\right]*\left[\begin{array}{c} \epsilon_{xx}\\ \epsilon_{yy}\\ \gamma_{xy}\\ \gamma_{xz}\\ \gamma_{yz} \end{array}\right]$$

The *Q*[*ij*] constants are derived from the modulus of elasticity *E*(*z*) as well as the Poisson ratio υ(z) utilizing the formulas in Equations (26)–(28)

(26) $$Q_{11}=Q_{22}=\frac{E\left(z\right)}{{1-\upsilon \left(z\right)}^{2}}$$

(27) $$Q_{12}=Q_{21}=\upsilon \left(z\right)\frac{E\left(z\right)}{{1-\upsilon \left(z\right)}^{2}}$$

(28) $$Q_{33}=Q_{44}=Q_{55}=\frac{E\left(z\right)}{2\left(1+\upsilon \left(z\right)\right)}$$

### 2.6. Derivation of the Governing Equation of the Plate

The strain energy (*U*) of the plate may be articulated using strain ε and stress σ as follows:

(29) $$U=\oint \left(\varepsilon^{T}\sigma \right)\partial V=∭{\varepsilon_{o}}^{T}{\left[H\right]}^{T}*\left[Q\right]*[H{]\varepsilon }_{o}\partial z\partial x\partial y$$

Equation (30) simplifies the calculation of [*D*], the material rigidity matrix that correlates the influence of material property variations throughout the thickness to the mid-plane. It is articulated as follows:

(30) $$D=\int_{-\frac{h}{2}}^{\frac{h}{2}}{\left[H\right]}^{T}*[Q]*[H]\partial z$$

The critical buckling load along the *x*- and *y*-axes is denoted as *N*_x_ and *N*_y_, respectively, while *N*_xy_ signifies shear buckling. The total work performed by external compressive pressures exerted on the edges of the plate is as follows:

(31) $$V\;=\;\frac{1}{2}∭\left\{N_{x}{\frac{\partial w}{\partial x}}^{2}\;+\;N_{y}{\frac{\partial w}{\partial y}}^{2}\;+\;2N_{xy}\frac{\partial w}{\partial x}\frac{\partial w}{\partial y}\right\}dzdxdy$$

Equation (31) can be reformulated as Equation (32), where *N* represents the compressive force matrix and [*Z_b_*] denotes the thickness coordinate matrix for buckling, both delineated in the Appendix A. The buckling strain vector *ϵ_b_* is defined as *ϵ_b_* = {∂w/∂x, ∂w/∂y}.

(32) $$V\;=\;\frac{1}{2}\iint {\epsilon_{b}}^{T}\left\{\int {\left[Z_{b}\right]}^{T}\hat{N}\left[Z_{b}\right]dz\right\}\epsilon_{b}\;\partial xdy$$

The geometric rigidity matrix [*D*] is derived via the compressive stress matrix [*N*] and the thickness coordinate matrix [*Z_b_*] provided in the Appendix A. This matrix facilitates the application of the suggested HSDT as a comparable single-layer theory to convert the three-dimensional domain into a two-dimensional domain for analytical purposes.

(33) $$\left[D_{G}\right]\;=\;\int_{z=-\frac{h}{2}}^{z\;=\;\frac{h}{2}}{\left[Z_{b}\right]}^{T}\left[\hat{N}\right]\left[Z_{b}\right]\partial z$$

The utilization of the virtual work principle concerning the strain energy, *U*, accumulated as a result of internal strains in the composite laminate, alongside the work necessary to impose the artificial constraint, *C*, as well as the external work induced by compressive stresses on the plate, *V*, results in the subsequent governing equation.

(34) $$\oint \delta \left(U\;+\;C^{*}-V\right)\partial \Omega \;=\;0$$

### 2.7. Finite Element Formulation

The formulation of the isoparametric finite element method for analyzing functionally graded material plates utilized nine-noded isoparametric Lagrangian shape functions. The employment of a nine-node element facilitates the implementation of a complete integration strategy. The shape functions utilized in the formulation are specified in Equation (35).

(35) $$N(\xi ,\eta )=\left\{\begin{array}{c} \frac{1}{4}\left(\xi -1\right)\left(\eta -1\right)\xi \eta ,\frac{1}{4}\left(\xi +1\right)\left(\eta -1\right)\xi \eta ,\frac{1}{4}\left(\xi +1\right)\left(\eta +1\right)\xi \eta \\ \frac{1}{4}\left(\xi -1\right)\left(\eta +1\right)\xi \eta ,-\frac{1}{2}\left(1-\xi^{2}\right)\left(1-\eta \right)\eta ,-\frac{1}{2}\left(1+\xi \right)\xi \left(\eta^{2}-1\right),\\ -\frac{1}{2}\left(\xi^{2}-1\right)\left(1+\eta \right)\eta ,-\frac{1}{2}\left(\xi -1\right)\xi \left(\eta^{2}-1\right),\left(1-\xi^{2}\right)\left(1-\eta^{2}\right)\;\;\; \end{array}\right\}$$

The midplane displacements at each node can be expressed as a linear combination of the nodal unknowns (*X*_i_) and isoparametric shape functions (*N*_i_) associated with the element, as described below:

(36) $$X=\sum_{i=1}^{9}N_{i}*X_{i}$$

Estimation of the mid-surface strain utilizing the nodal unknowns at each element node as outlined in Equation (36) can be performed via differentiation of the isoparametric shape functions with the aid of the Jacobian matrix (*J*), i.e., [*J*] = ∂(ξ, η)/∂(x,y). The operator matrix [*B*], which includes the shape function derivative for the midplane strain vector, can be found in Appendix A. The equation, *ε_o_* = [*B*]×{*X*} is used to derive the midplane strain vector from the operator matrix.

The midplane strain vector is featured in Equation (37), which applies virtual work principle to equate the internal virtual work of the plate, *U*, with the buckling energy induced by the external compressive forces, *V*, exerted on the plate.

(37) $$\iint {\delta X_{o}}^{T}*\left\{{\left[B\right]}^{T}\left[D\right]\left[B\right]*J*\partial \xi \partial \eta *X-\left\{B_{c}\hat{N}B_{c}\right\}*J*\partial \xi \partial \eta \right\}=0$$

Due to the arbitrary selection of virtual displacements, the formula for geometric stiffness matrix, [*K_G_*], can be extracted from Equation (38) as demonstrated below:

(38) $${\left[K_{G}\right]}_{e}=\iint \left\{B_{c}\hat{N}B_{c}\right\}*J*\partial \xi \partial \eta$$

## 3. Results

The thickness ratio (a/h) is varied along with variation in the mesh size. The characteristics of the laminated composite material are the following: -(E_1_ = 40 E_2,_ G_12_ = G_13_ = 0.6 E_2_, G_23_ = 0.5 E_2_, υ_12_ = 0.25). The non-dimensional buckling load, N_cr_ = Na^2^/E_2_h^3^, is taken for calculations. The buckling analysis is performed for square, simply supported cross-ply laminated rectangular plates under biaxial compressive loading and Table 1 presents the mesh convergence results. The findings were compared with the existing studies by Anish et al. [52], Vescovini and Dozio [53], and Georgantzinos et al. [51] and the convergence of the values of critical buckling load have been found satisfactory.

Table 2 shows the impact of changes in the modular ratio (E_1_/E_2_) for various geometric configuration of the laminate. The properties of the laminated composite material are the following: -(G_12_ = G_13_ = 0.6 E_2_, G_23_ = 0.5 E_2_, υ_12_ = 0.25). The non-dimensional buckling loads, N_cr_ = Na^2^/E_2_h^3^, is taken for calculations. Buckling analysis is carried out using a square laminated composite plate of moderate thickness (a/h = 10), which is simply supported on all its sides. It was found that critical buckling load of the laminated composite increases along with modular ratio. The result of the analysis is validated using existing results like Georgantzinos et al. [51]), (Anish et al. [52]), and presented in Table 2.

The effect of MWCNT reinforcements on the critical buckling load of a square composite laminate was studied and Table 3 shows the obtained results. The T300/BSL914C fiber-polymer composite is utilized as the analyzing material. Various composite laminate schemes and thickness ratios (a/h) are taken into consideration. The plate has simply supported boundary conditions. N_cr_ (MN/m) represents the critical buckling stress. It was found that increasing MWCNT reinforcement decreases the critical buckling load. The increase in critical buckling load is more significant for lower fractions of MWCNT up to 2%.

The impact of MWCNT reinforcements on the critical buckling load of a square composite laminate was studied and Table 4 shows the obtained results. The MWCNT-added composite laminate is clamped on all sides. The critical buckling load corresponds to N_cr_ (MN/m). With fixed boundary conditions, a rise in the uniaxial critical buckling load is observed. The uniaxial critical buckling load decreases as MWCNT reinforcement increases from 0.5% to around 5 to 8%.

The impact of MWCNT reinforcements on the critical buckling load of a square composite laminate was studied and Table 5 shows the obtained results. The plate is supported on two sides and restrained on two others. N_cr_ (MN/m) is the notation for the critical buckling load. The critical buckling load of uniaxial buckling rises under constrained boundary conditions. Critical buckling load decreases as MWCNT reinforcement increases and reaches optimum at 8%.

In determining the critical buckling load of uni- and biaxial buckling, the structural configuration of the laminated composite structure is crucial. As the number of layers increases, the influence of symmetric/anti-symmetric layer schemes is studied; Table 6 and Table 7 show the results obtained for uni- and biaxial buckling, respectively. The plate’s geometry is square, its thickness is moderate (a/h = 10), and its boundary conditions are simply supported. The antisymmetric composite laminate is configured as follows:—AS (Type 1) [0°/90°/0°/90°], AS (Type 2) [0°/90°/0°/90°/0°/90°], AS (Type 3) [0°/90°/0°/90°/0°/90°/0°/90°], AS (Type 4) [0°/90°/0°/90°/0°/90°/0°/90°/0°/90°]. For the symmetric composite laminate, the composite laminate is configured as follows:—SYM (Type 1) [0°/90°]_2_, SYM (Type 2) [0°/90°/0°]_2_, SYM (Type 3) [0°/90°/0°/90°]_2_ and SYM (Type 4) [0°/90°/0°/90°/0°]_2_. Compared to symmetric composite laminates, the magnitude of critical load (N_cr_ in MN/m) of uni- and biaxial buckling is greater for anti-symmetric composite laminates as presented in Table 6. Similar trends are observed in Table 7 with much lower critical buckling loads.

The diversity in values of the critical buckling load (N_cr_ in MN/m) of uniaxial and biaxial buckling for various laminated composite schemes is studied and the results are presented in Table 8 and Table 9. The analysis is performed on a square composite laminate that is simply supported and reinforced with MWCNT fibers. It has been found that the critical buckling load increases with core thickness. The addition of MWCNT fibers reduces the critical buckling load and the optimal volume of MWCNT reinforcement achieved is 8%.

The effect of the randomness of MWCNT reinforcement fiber orientation factor (f_r_) on the critical buckling load of uni- and biaxial buckling is studied for different volumes of MWCNT reinforcement and different fiber orientations in the laminates. The laminated composite is composed of AS40/3501-6 fiber-polymer composite with MWCNT reinforcement and α = 10, β = 0.9, and f_w_ = 0.6. The uniaxial and biaxial buckling analysis is performed on a square composite laminate with the following fiber orientations [θ°/−θ°/θ°/−θ°] and the results are shown in Table 10 for uniaxial buckling and Table 11 for biaxial buckling. N_cr_ (MN/m) denotes the frequency of the critical buckling load. Clearly, the random orientation of the fibers will increase the critical buckling load of uniaxial and biaxial buckling.

The influence of the MWCNT reinforcement waviness factor (f_w_) on the critical buckling load (N_cr_ in MN/m) of uniaxial and biaxial buckling is investigated for various MWCNT reinforcement volumes and fiber orientations in the composite laminate. The results of the buckling analysis conducted on the square composite laminate with the following fiber orientations [θ°/−θ°/θ°/−θ°] are shown in Table 12 for uniaxial buckling and Table 13 for biaxial buckling. It can be seen that the random orientation of fibers will marginally reduce the critical buckling load of uni- and biaxial buckling. In addition, it was found that the optimal value of is 5% when f_w_ = 0.2, but 8% when f_w_ = 0.6 and f_w_ = 1.0.

It is necessary to investigate the effect of fiber agglomeration (f_a_), as greater agglomeration results in a more rigid polymer matrix, which in turn impacts the critical buckling load of uni- and biaxial buckling for the analysis. For the analysis, AS40/3501-6 fiber-polymer composite is utilized as the material. The plate has a moderate thickness (a/h = 10), being simply supported on all of its square sides. The orientation and arrangement of the fibers are determined by the critical buckling load of the uniaxial and biaxial buckling, which can be calculated using N_cr_ (MN/m). The resulting values are shown in Table 14 for uniaxial buckling and Table 15 for biaxial buckling. The increased agglomeration results in decrease of critical buckling load of uni- and biaxial buckling.

**Figure 2:**
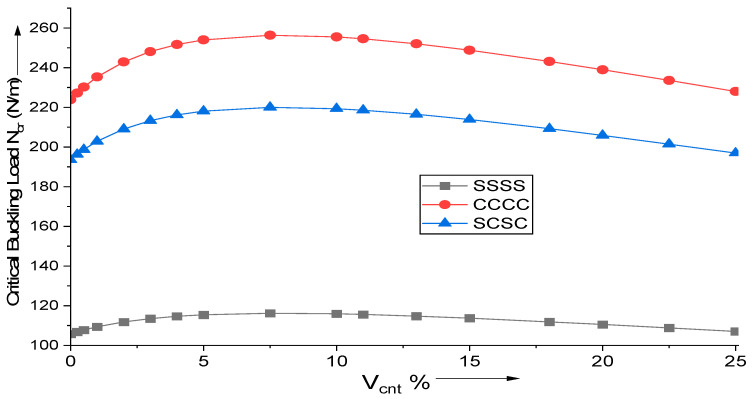
Influence of MWCNT reinforcement addition on the critical buckling load for uniaxial buckling of (AS40/3501-6) laminated composite.

Parametric analysis is performed for various boundary conditions, on a square, moderately thick (a/h = 10) plate. Various percentages of MWCNT reinforcement are added and their impact on the critical buckling load for uniaxial bucking, N_cr_ (MN/m), is observed. The optimal MWCNT reinforcement volume is between 8 and 10 percent. In the case of simply supported boundary constraints, the critical buckling load is the least, whereas for plates with all sides clamped it is the largest. Figure 2 depicts the variation of critical buckling load with the addition of MWCNT reinforcements.

**Figure 3:**
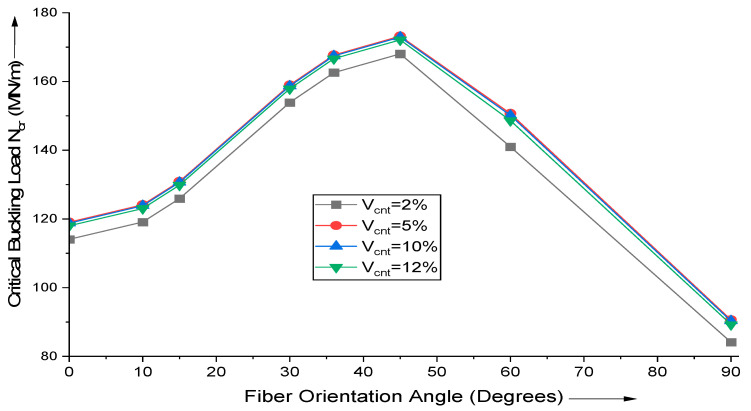
Effect of variation in fiber orientation angle on the critical buckling load of uniaxial buckling of (AS40/3501-6) laminated composite.

For various MWCNT fiber additions, the influence of changes in fiber orientation on the critical buckling load of uniaxial buckling is investigated. The plate is supported by a simple base and is moderately thick (a/h = 10). Figure 3 depicts the findings of a paramedic research conducted on a square, simply supported composite laminate with layer configuration [θ°/−θ°/θ°/−θ°]. Using the proposed HSDT-based FE (finite element) formulation, the critical buckling load given by N_cr_ (MN/m) is computed. Observations indicate that the magnitude of critical buckling load is greatest for (θ = 45°) cross-ply laminate.

**Figure 4:**
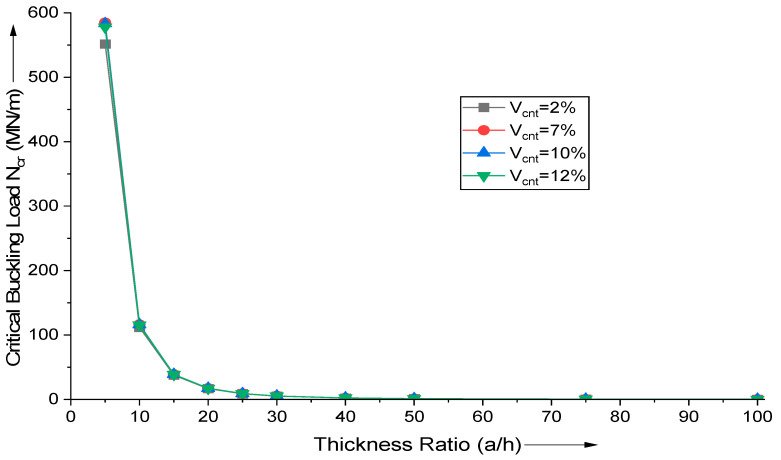
Influence of variation in thickness ratio (a/h) on the critical buckling load of uniaxial buckling of (AS40/3501-6) laminated composite.

The influence of the aspect ratio (a/h) and MWCNT addition on critical buckling load is investigated. Using the proposed HSDT-based FE formulation, the critical buckling load of uniaxial buckling, N_cr_ (MN/m), is calculated. As shown in Figure 4, when the thickness ratio increases, the variation of the critical buckling load decreases as after (a/h ratio > 40), there is no variation of significance. This demonstrates that the thickness of the plate significantly influences the critical buckling load of uniaxial buckling for thick plates but it is negligible for thin plates.

**Figure 5:**
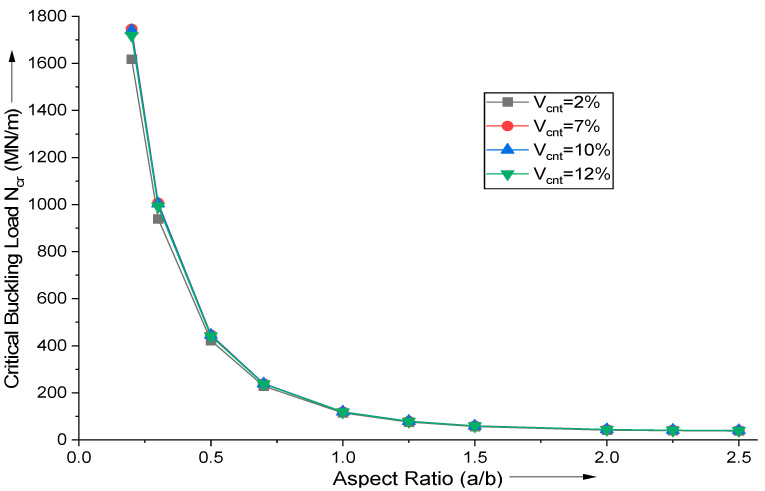
Influence of variation in aspect ratio (a/b) on the critical buckling load for uniaxial buckling of (AS40/3501-6) laminated composite.

The aspect ratio (a/b) has a significant influence on the critical buckling load of uniaxial buckling. The influence of aspect ratio and MWCNT addition is explored in Figure 5. A high aspect ratio contributes to a very high critical buckling load since the decrease in smaller side increases the plate’s stiffness, thereby raising the critical buckling load. Using the proposed HSDT-based FE formulation, the critical buckling load for uniaxial buckling, N_cr_ (MN/m), is estimated. Figure 5 demonstrates that increasing aspect ratio (a/b) raises the critical buckling load, since the smaller dimensions control the phenomenon of buckling.

## 4. Conclusions

A comprehensive hybrid polynomial and hyperbolic form function-based two-dimensional mathematical model is introduced to analyze the instability of MWCNT-reinforced composite plates. The 2D finite element implementation of the current model with a nine-node isoparametric finite element is executed in proprietary MATLAB (R2019a) code. The model was evaluated to examine the impact of including MWCNT fillers into laminated composites under various external loading, thickness ratios, aspect ratios, laminate structures, and support conditions. The convergence and correctness of the finite element model were validated against available research results and comprehensive parametric investigations were carried out to create standards for future research. The following conclusions were drawn from the present research:

- The current 2D finite element model has sufficient efficiency to yield results that are closely matching with benchmark outcomes of established 3D theories. For moderately thick and thin plates, the finite element model exhibits exceptional performance.
- When MWCNT fillers are incorporated in the polymer matrix, the critical buckling load of the composite laminated significantly increases.
- MWCNT incorporation enhances the plate performance up to 8–10% by volume. Beyond that value, the composite exhibits deterioration of its elastic characteristics.
- The critical buckling stresses of the laminated structure with cross-ply orientation is increased, compared to alternative orientations of carbon fiber reinforcement within the composite.
- The optimization of the critical buckling load in the composite plate with the MWCNT inclusion being particularly beneficial for plates that are fixed on all sides.
- The augmentation of laminate layers increases the critical buckling load of the laminate. The impact of the number of layers is particularly significant in thick plates.

## Figures and Tables

**Table 1:** The mesh convergence study for the non-dimensional critical buckling load under biaxial compression.

Mesh Size	a/h		
	10	20	50
2 × 2	5.0711	5.9981	6.5621
5 × 5	4.8601	5.5165	5.7837
7 × 7	4.8538	5.4977	5.7324
9 × 9	4.8523	5.4927	5.7162
11 × 11	4.8518	5.491	5.7098
13 × 13	4.8516	5.4902	5.7069
Anish et al. [52]	4.8441	5.489	5.7084
Vescovini and Dozio [53]	4.9095	5.5082	5.7063
Georgantzinos et al. [51]	4.7421	5.4192	5.6901

**Table 2:** Changes in non-dimensional critical buckling load of the composite laminate with different modular ratios.

Scheme	Source	E1/E2				
		3	10	20	30	40
0°/90°/0°	[51]	4.9945	9.486	14.567	18.5485	21.764
	[52]	5.3142	9.6982	14.6927	18.6343	21.8415
	Present	5.3138	9.7044	14.7138	18.6714	21.8935
0°/90°/90°/0°	[51]	4.997	9.58	14.95	19.3035	22.919
	[52]	5.3197	9.8087	15.1025	19.4295	23.0565
	Present	5.3179	9.8144	15.1228	19.462	23.0956

**Table 3:** The effect of addition of MWCNT fiber reinforcements on the critical buckling load of square simply supported MWCNT-added composite laminate.

Laminate		MWCNT Fraction Addition					
Scheme	a/h	0.50%	1%	2%	5%	8%	10%
0°/90°	5	543.791	553.6757	569.2	593.9374	599.6862	597.7721
	10	101.4447	103.2109	105.8804	109.8822	110.7819	110.4833
	20	14.8284	15.0385	15.3586	15.8437	15.9536	15.9171
0°/90°/0°	5	503.1055	516.8275	537.5006	568.2719	575.1457	572.8662
	10	111.5892	113.5301	116.4347	120.7275	121.6831	121.3663
	20	17.6615	17.8562	18.1516	18.5973	18.698	18.6645
0°/90°/90°/0°	5	537.8558	551.5335	572.0962	602.6163	609.4214	607.1652
	10	115.5143	117.3682	120.1461	124.2597	125.1769	124.8728
	20	17.8619	18.0498	18.3356	18.7685	18.8666	18.834

**Table 4:** The influence of adding MWCNT fiber reinforcements on the critical buckling load of square clamped MWCNT-added composite laminate.

Plate		MWCNT Fraction Addition					
Arrangement	a/h	0.50%	1%	2%	5%	8%	10%
0°/90°	5	562.8578	580.0492	606.0517	644.9657	653.6906	650.796
	10	152.6201	156.1521	161.4829	169.4514	171.2388	170.6457
	20	27.0236	27.6108	28.559	30.1824	30.5918	30.4539
0°/90°/0°	5	656.9827	679.5254	713.6846	764.888	776.3731	772.5628
	10	228.5455	234.3272	242.9459	255.5727	258.361	257.4377
	20	51.23	51.9552	53.0244	54.5709	54.91	54.7978
0°/90°/90°/0°	5	664.0645	686.8188	721.2755	772.8762	784.4428	780.6058
	10	237.5133	243.4137	252.1796	264.9595	267.7718	266.8409
	20	52.6417	53.3298	54.3443	55.8126	56.1347	56.0281

**Table 5:** The effect of addition of MWCNT fiber reinforcements on the critical buckling load of square MWCNT-added composite laminate simply supported on two sides and clamped on the other two sides.

Plate		MWCNT Fraction Addition					
Arrangement	a/h	0.50%	1%	2%	5%	8%	10%
0°/90°	5	529.8289	545.1978	568.3805	602.9546	610.69	608.1243
	10	124.431	127.0384	131.012	137.1732	138.6487	138.1521
	20	19.8434	20.1787	20.6886	21.4596	21.634	21.576
0°/90°/0°	5	606.8153	627.0582	657.7202	703.6563	713.9561	710.5392
	10	203.3076	208.2967	215.7274	226.5938	228.9894	228.1963
	20	46.3969	47.01	47.9088	49.1977	49.4785	49.3857
0°/90°/90°/0°	5	624.5203	645.1995	676.4719	723.2088	733.6694	730.1999
	10	205.9699	210.6877	217.7475	228.09	230.3702	229.6153
	20	44.9756	45.532	46.349	47.5239	47.7805	47.6957

**Table 6:** Influence of fiber orientation on the critical buckling load for uniaxial buckling of (T300/BSL914C) laminated composite.

Laminate	Layer	MWCNT Fraction Addition					
Scheme	Number	0.50%	1%	2%	5%	8%	10%
AS (Type 1)	4	110.6392	112.2191	114.5952	118.1367	118.9285	118.6712
AS (Type 2)	6	112.3899	113.9697	116.3443	119.8830	120.6738	120.4163
AS (Type 3)	8	112.9148	114.4960	116.8718	120.4113	121.2018	120.9452
AS (Type 4)	10	113.1448	114.7261	117.1032	120.6426	121.4340	121.1775
SYM (Type 1)	4	107.9615	109.6819	112.2584	116.0776	116.9278	116.6517
SYM (Type 2)	6	111.2860	112.9265	115.3885	119.0470	119.8627	119.5971
SYM (Type 3)	8	112.1737	113.7924	116.2212	119.8327	120.6388	120.3772
SYM (Type 4)	10	112.6803	114.2857	116.6966	120.2816	121.0831	120.8228

**Table 7:** Effect of fiber orientation laminate schemes on the critical buckling load for biaxial buckling of (T300/BSL914C) laminated composite.

Laminate	Layer	MWCNT Fraction Addition					
Scheme	Number	0.50%	1%	2%	5%	8%	10%
AS (Type 1)	4	55.3315	56.1213	57.3089	59.0783	59.4743	59.3462
AS (Type 2)	6	56.1972	56.9872	58.1741	59.9425	60.3385	60.2097
AS (Type 3)	8	56.4583	57.2485	58.4369	60.2062	60.6014	60.4737
AS (Type 4)	10	56.5728	57.3635	58.5521	60.3213	60.7170	60.5887
SYM (Type 1)	4	53.9812	54.8405	56.1297	58.0383	58.4639	58.3253
SYM (Type 2)	6	55.6430	56.4637	57.6947	59.5230	59.9313	59.7991
SYM (Type 3)	8	56.0873	56.8957	58.1101	59.9164	60.3194	60.1886
SYM (Type 4)	10	56.3402	57.1428	58.3483	60.1413	60.5410	60.4114

**Table 8:** Influence of core thickness on the critical buckling load for uniaxial buckling of (T300/BSL914C) laminated composite.

Laminate	a/h	MWCNT Fraction Addition					
Scheme		0.50%	1%	2%	5%	8%	10%
0°/90°/90°/0°	5	515.1120	528.0637	547.5149	576.3523	582.7663	580.6688
	10	107.9615	109.6819	112.2584	116.0776	116.9278	116.6517
	20	16.4544	16.6313	16.9002	17.3075	17.3995	17.3697
	50	1.1250	1.1349	1.1502	1.1738	1.1792	1.1774
0°/90°/… 90°/90°/0°	5	455.0452	474.0221	503.0560	541.4142	549.0997	546.6077
	10	93.9497	95.6678	98.2568	102.1262	102.9933	102.7101
	20	13.6428	13.8485	14.1606	14.6316	14.7378	14.7033
	50	0.9199	0.9327	0.9522	0.9818	0.9885	0.9863
0°/0°/0°/90°/ 90°/90°/90°/ 0°/0°/0°	5	494.1663	507.1700	526.7186	555.7527	562.2220	560.1037
	10	105.7326	107.5040	110.1570	114.0823	114.9556	114.6712
	20	16.3446	16.5253	16.7995	17.2139	17.3073	17.2771
	50	1.1236	1.1336	1.1490	1.1726	1.1780	1.1763

**Table 9:** Influence of core thickness on the critical buckling load for biaxial buckling of (T300/BSL914C) laminated composite.

Laminate	a/h	MWCNT Fraction Addition					
Scheme		0.50%	1%	2%	5%	8%	10%
0/90/90/0	5	257.5120	263.9904	273.7220	288.1510	291.3620	290.3091
	10	53.9812	54.8405	56.1297	58.0383	58.4639	58.3253
	20	8.2272	8.3156	8.4501	8.6537	8.6998	8.6848
	50	0.5625	0.5675	0.5751	0.5869	0.5896	0.5887
0/90/90990/0	5	268.8937	275.3691	285.0769	299.4343	302.6181	301.5755
	10	55.1978	56.0299	57.2764	59.1275	59.5401	59.4064
	20	8.2867	8.3731	8.5047	8.7044	8.7496	8.7350
	50	0.5632	0.5682	0.5758	0.5875	0.5902	0.5893
0/0/0/90/90/… 90/90/0/0/0	5	247.0685	253.5699	263.3436	277.8638	281.0983	280.0392
	10	52.8663	53.7520	55.0785	57.0411	57.4778	57.3351
	20	8.1723	8.2627	8.3998	8.6069	8.6536	8.6385
	50	0.5618	0.5668	0.5745	0.5863	0.5890	0.5881

**Table 10:** Effect of spatial randomness of MWCNT reinforcements on critical buckling load for uniaxial buckling of (AS40/3501-6) laminated composite.

	Theta	MWCNT Fiber Volume Addition					
		0.50%	1%	2%	5%	8%	10%
f_r_ = 1/3	0°	104.5029	107.4708	111.8107	118.3096	120.1939	120.1508
	15°	115.0004	117.9738	122.2991	128.7349	130.5956	130.5525
	30°	140.4115	143.3902	147.6498	153.8607	155.6359	155.5928
	45°	153.2965	156.3349	160.6446	166.8549	168.6145	168.5724
f_r_ = 1/6	0°	102.8604	104.5949	107.2012	111.0602	111.9009	111.5909
	15°	113.3483	115.0924	117.7043	121.5541	122.3891	122.0804
	30°	138.7396	140.5041	143.1209	146.9204	147.7385	147.4380
	45°	151.5834	153.3905	156.0598	159.9100	160.7341	160.4310

**Table 11:** Effect of spatial randomness of MWCNT reinforcements on critical buckling load for biaxial buckling of (AS40/3501-6) laminated composite.

	Theta	MWCNT Fraction Addition					
		0.50%	1%	2%	5%	8%	10%
f_r_ = 1/3	0°	52.2543	53.7374	55.9069	59.1554	60.0981	60.0754
	15°	57.5012	58.9874	61.1501	64.3674	65.2978	65.2757
	30°	70.2058	71.6951	73.8254	76.9298	77.8173	77.7964
	45°	76.6190	78.1463	80.3100	83.4210	84.3012	84.2801
f_r_ = 1/6	0°	51.4339	52.3008	53.6031	55.5322	55.9520	55.7971
	15°	56.6756	57.5467	58.8527	60.7770	61.1945	61.0413
	30°	69.3698	70.2525	71.5605	73.4602	73.8692	73.7190
	45°	75.7552	76.6664	78.0084	79.9412	80.3547	80.2027

**Table 12:** Effect of waviness of MWCNT reinforcements on the critical buckling load for uniaxial buckling of MWCNT-added (AS40/3501-6) composite.

	Theta	MWCNT Fraction Addition					
		0.50%	1%	2%	5%	8%	10%
f_w_ = 0.2	0°	101.4327768	102.0222497	102.9035755	104.0306441	103.867248	103.3631445
	15°	111.9099204	112.5040129	113.3929287	114.5261536	114.3622272	113.8544212
	30°	137.2731372	137.8809619	138.7854487	139.9337788	139.7678256	139.2541065
	45°	150.0780276	150.7026596	151.6303944	152.8075341	152.6377544	152.1107955
f_w_ = 0.6	0°	102.860415	104.594931	107.2011507	111.0601908	111.9008766	111.5909345
	15°	113.3483085	115.0923678	117.7043007	121.5540504	122.389068	122.080399
	30°	138.739608	140.5040802	143.1209234	146.9203956	147.7384824	147.438041
	45°	151.583376	153.3905292	156.0598039	159.909966	160.7341362	160.431001
f_w_ = 1.0	0°	104.0146076	106.6153063	110.4537868	116.1971482	117.7632876	117.6366155
	15°	114.5101062	117.1191211	120.9502798	126.647136	128.1964128	128.0707962
	30°	139.9184072	142.5380357	146.3288876	151.861201	153.3461536	153.2266022
	45°	152.7915022	155.4665703	159.3113368	164.8640058	166.3437676	166.2253229

**Table 13:** Effect of waviness of MWCNT reinforcements on the critical buckling load for biaxial buckling of MWCNT-added (AS40/3501-6) composite.

	Theta	MWCNT Fraction Addition					
		0.50%	1%	2%	5%	8%	10%
f_w_ = 0.2	0°	50.7210	51.0157	51.4555	52.0187	51.9374	51.6849
	15°	55.9563	56.2534	56.6969	57.2636	57.1821	56.9282
	30°	68.6361	68.9405	69.3923	69.9669	69.8839	69.6266
	45°	74.9956	75.3112	75.7792	76.3723	76.2871	76.0220
f_w_ = 0.6	0°	51.4339	52.3008	53.6031	55.5322	55.9520	55.7971
	15°	56.6756	57.5467	58.8527	60.7770	61.1945	61.0413
	30°	69.3698	70.2525	71.5605	73.4602	73.8692	73.7190
	45°	75.7552	76.6664	78.0084	79.9412	80.3547	80.2027
f_w_ = 1.0	0°	52.0111	53.3101	55.2290	58.0991	58.8828	58.8189
	15°	57.2555	58.5601	60.4751	63.3236	64.0982	64.0354
	30°	69.9592	71.2685	73.1644	75.9300	76.6731	76.6133
	45°	76.3648	77.7110	79.6410	82.4246	83.1649	83.1051

**Table 14:** Effect of agglomeration of MWCNT reinforcements on the critical buckling load for uniaxial buckling of (AS40/3501-6) laminated composite.

	Theta	MWCNT Fraction Addition					
		0.50%	1%	2%	5%	8%	10%
a = 5	0°	102.9502	104.8738	108.0614	114.3211	117.8202	119.2519
	15°	113.4395	115.3727	118.5643	124.7908	128.2520	129.6663
	30°	138.8334	140.7875	143.9758	150.0709	153.4001	154.7497
	45°	151.6810	153.6811	156.9293	163.0768	166.3983	167.7373
a = 10	0°	102.8604	104.5949	107.2012	111.0602	111.9009	111.5909
	15°	113.3483	115.0924	117.7043	121.5541	122.3891	122.0804
	30°	138.7396	140.5041	143.1209	146.9204	147.7385	147.4380
	45°	151.5834	153.3905	156.0598	159.9100	160.7341	160.4310
a = 15	0°	102.7797	104.3396	106.4182	108.3463	107.4039	106.1421
	15°	113.2672	114.8365	116.9208	118.8490	117.9065	116.6444
	30°	138.6576	140.2474	142.3399	144.2588	143.3226	142.0627
	45°	151.4989	153.1284	155.2649	157.2157	156.2645	154.9830

**Table 15:** Effect of agglomeration of MWCNT reinforcements on the critical buckling load for biaxial buckling of (AS40/3501-6) laminated composite.

	Theta	MWCNT Fraction Addition					
		0.50%	1%	2%	5%	8%	10%
a = 5	0°	51.4793	52.4398	54.0327	57.1617	58.9107	59.6272
	15°	56.7207	57.6873	59.2827	62.3954	64.1266	64.8332
	30°	69.4167	70.3937	71.9879	75.0355	76.7006	77.3748
	45°	75.8045	76.8126	78.4459	81.5278	83.1916	83.8627
a = 10	0°	51.4339	52.3008	53.6031	55.5322	55.9520	55.7971
	15°	56.6756	57.5467	58.8527	60.7770	61.1945	61.0413
	30°	69.3698	70.2525	71.5605	73.4602	73.8692	73.7190
	45°	75.7552	76.6664	78.0084	79.9412	80.3547	80.2027
a = 15	0°	51.3940	52.1727	53.2120	54.1757	53.7040	53.0745
	15°	56.6345	57.4192	58.4609	59.4250	58.9538	58.3227
	30°	69.3283	70.1242	71.1695	72.1294	71.6608	71.0314
	45°	75.7130	76.5341	77.6092	78.5900	78.1117	77.4674

## Data Availability

The original contributions presented in this study are included in the article. Further inquiries can be directed to the corresponding author.

## Appendix A

(A1)
$${\left[B\right]}^{T}=\sum_{i=1}^{9}\left[\begin{array}{cccccccccccccccccc} N_{i,x} & 0 & N_{i,y} & 0 & 0 & 0 & 0 & 0 & 0 & 0 & 0 & 0 & 0 & 0 & 0 & 0 & 0 & 0\\ 0 & N_{i,y} & N_{i,x} & 0 & 0 & 0 & 0 & 0 & 0 & 0 & 0 & 0 & 0 & 0 & 0 & 0 & 0 & 0\\ 0 & 0 & 0 & N_{i,x} & N_{i,y} & 0 & 0 & 0 & 0 & 0 & 0 & 0 & 0 & 0 & 0 & 0 & 0 & 0\\ 0 & 0 & 0 & 0 & 0 & N_{i,x} & 0 & N_{i,y} & N_{i} & 0 & 0 & 0 & 0 & 0 & 0 & 0 & 0 & 0\\ 0 & 0 & 0 & 0 & 0 & 0 & N_{i,y} & N_{i,x} & 0 & N_{i} & 0 & 0 & 0 & 0 & 0 & 0 & 0 & 0\\ 0 & 0 & 0 & N_{i} & 0 & 0 & 0 & 0 & 0 & 0 & N_{i,x} & 0 & N_{i,y} & N_{i} & 0 & 0 & 0 & 0\\ 0 & 0 & 0 & 0 & 0 & 0 & 0 & 0 & 0 & 0 & 0 & N_{i,y} & N_{i,x} & 0 & N_{i} & 0 & 0 & 0\\ 0 & 0 & 0 & 0 & 0 & 0 & 0 & 0 & 0 & 0 & 0 & 0 & 0 & 0 & 0 & N_{i} & N_{i,x} & N_{i,y} \end{array}\right]$$

(A2)
$$\left[H\right]=\left[\begin{array}{cccccccccccccccccc} 1 & 0 & 0 & 0 & 0 & -z & 0 & 0 & 0 & 0 & -f\left(z\right) & 0 & 0 & 0 & 0 & 0 & 0 & 0\\ 0 & 1 & 0 & 0 & 0 & 0 & -z & 0 & 0 & 0 & 0 & -f\left(z\right) & 0 & 0 & 0 & 0 & 0 & 0\\ 0 & 0 & 0 & 0 & 0 & 0 & 0 & 0 & 0 & 0 & 0 & 0 & 0 & 0 & 0 & g^{\prime }\left(z\right) & 0 & 0\\ 0 & 0 & 1 & 0 & 0 & 0 & 0 & -z & 0 & 0 & 0 & 0 & -f\left(z\right) & 0 & 0 & 0 & 0 & 0\\ 0 & 0 & 0 & 1 & 0 & 0 & 0 & 0 & -1 & 0 & 0 & 0 & 0 & -f^{\prime }\left(z\right) & 0 & 0 & g^{\prime }\left(z\right) & 0\\ 0 & 0 & 0 & 0 & 1 & 0 & 0 & 0 & 0 & -1 & 0 & 0 & 0 & 0 & -f^{\prime }\left(z\right) & 0 & 0 & g^{\prime }\left(z\right) \end{array}\right]$$

(A3)
$$N(\xi ,\eta )=\left\{\begin{array}{c} \frac{1}{4}\left(\xi -1\right)\left(\eta -1\right)\xi \eta ,\frac{1}{4}\left(\xi +1\right)\left(\eta -1\right)\xi \eta ,\frac{1}{4}\left(\xi +1\right)\left(\eta +1\right)\xi \eta ,\\ \frac{1}{4}\left(\xi -1\right)\left(\eta +1\right)\xi \eta ,-\frac{1}{2}\left(1-\xi^{2}\right)\left(1-\eta \right)\eta ,-\frac{1}{2}\left(1+\xi \right)\xi \left(\eta^{2}-1\right),\\ -\frac{1}{2}\left(\xi^{2}-1\right)\left(1+\eta \right)\eta ,-\frac{1}{2}\left(\xi -1\right)\xi \left(\eta^{2}-1\right),\left(1-\xi^{2}\right)\left(1-\eta^{2}\right) \end{array}\right\}$$
